# Editorial: State of the art, opportunities and challenges in the use of medical detection dogs in the laboratory and in the field

**DOI:** 10.3389/fmed.2023.1283474

**Published:** 2023-09-05

**Authors:** Holger A. Volk, Guillaume Alvergnat, Anne-Lise Chaber, Dominique Grandjean, Fernando O. Mardones

**Affiliations:** ^1^Department of Small Animal Medicine and Surgery, University of Veterinary Medicine Hannover, Hannover, Germany; ^2^International Affairs Bureau, Ministry of Interior of the United Arab Emirates, Dubai, United Arab Emirates; ^3^School of Animal and Veterinary Sciences, University of Adelaide, Adelaide, SA, Australia; ^4^Global One Health Alliance Pty Ltd., West Lakes Shore, SA, Australia; ^5^École Nationale Vétérinaire d'Alfort, Université Paris-Est, Maisons-Alfort, France; ^6^Escuela de Medicina Veterinaria, Facultad de Agronomía e Ingeniería Forestal, Facultad de Ciencias Biológicas y Facultad de Medicina, Pontificia Universidad Católica de Chile, Santiago, Chile; ^7^Department of Pediatric Infectious Diseases and Immunology, Facultad de Medicina, Pontificia Universidad Católica de Chile, Santiago, Chile

**Keywords:** SARS-CoV-2, corona, diagnostic test, medical detection dogs, COVID

In the expansive realm of medical diagnostics, the remarkable olfactory capabilities of canines, combined with their proficiency in operant conditioning, offer a unique avenue for the utilization of medical detection dogs. While the use of scent detection dogs by law enforcement agencies and customs officials to detect substances like money, explosives, or drugs is well-established and widely accepted, the application of medical detection dogs in healthcare is still in its infancy. The recent emergence of research on the employment of medical detection dogs to identify individuals with infectious or non-infectious diseases, particularly during the SARS-CoV-2 pandemic, has garnered significant attention. However, skepticism from the medical profession persists, necessitating a critical exploration of the potential of canine scent detection as a medical test, particularly in the context of infectious diseases such as COVID-19.

The primary objective of this Research Topic is to critically examine the potential of canine scent detection as a medical test, focusing specifically on infectious diseases like COVID-19. By delving into the function and significance of canines' olfaction and evaluating their limitations, as well as their potentials, financial considerations, biosafety, ethical concerns, and animal welfare considerations, a collection of review and expert opinion articles shed light on the role that dogs can play as biomedical detectors for a range of infectious and non-infectious diseases (D'Aniello et al.; Maughan et al.; Meller et al.; Mutesa et al.; Singletary et al.).

One study within this Research Topic focuses on training dogs to detect SARS-CoV-2 positive samples and evaluates their ability to differentiate between SARS-CoV-2 infections and viral infections of a different origin (ten Hagen et al.). The findings reveal that dogs exhibit a mean diagnostic sensitivity of 73.8% and a specificity of 95.1% when presented with swab samples from individuals infected with viruses other than SARS-CoV-2. This demonstrates that dogs can indeed distinguish SARS-CoV-2 infections from other viral infections, although with lower diagnostic sensitivities compared to earlier studies, which used only negative controls as discriminators. The authors conclude that it's necessary to include samples from individuals with other respiratory infectious agents alongside negative and positive COVID-19 samples when training and evaluating the performance of COVID-19 scent detection dogs.

Another study presented in this Research Topic describes a field experience in Mexico where dogs were trained to detect COVID-19 using sweat and saliva samples from positive patients (Mancilla-Tapia et al.). The results indicate that four out of six dogs were able to detect positive samples, demonstrating promising sensitivity and specificity values. The authors suggest that with further exposure to sweat and saliva samples from COVID-19-positive individuals, the dogs' detection capacity could be enhanced, making them valuable allies in pandemic control.

A similar field study was conducted in Rwanda. During the Delta wave, the sensitivity of the dogs' COVID-19 detection ranged from 75.0 to 89.9%, and the specificity from 96.1 to 98.4% for the lowest- and highest-performing dogs, respectively (Mutesa et al.). However, these trained scent detection dogs performed worse during the Omicron wave, with a sensitivity of 36.6 to 41.5%, while specificity remained above 95% for all dogs. This highlights that dogs might need to be retrained for different strains of a virus or that dogs' scent detection performance varies depending on the viral strain. The Rwanda study also pointed out that medical scent detection dogs could be faster and more cost-effective than most antigen or PCR-based tests.

Interestingly, dogs trained to detect acute SARS-CoV-2 infections were also able to detect samples from Long COVID patients (Twele et al.). The dogs reliably detected these Long COVID samples when presented alongside negative samples, but not as reliably when presented alongside acute SARS-CoV-2 samples. The authors suggested that this could be attributed to a titration effect, with Long COVID samples having a lower content of volatile organic compounds (VOCs) than acute SARS-CoV-2 samples.

In another contribution to this Research Topic, researchers explored the use of detection dogs to identify restricted and hazardous biological agents through their volatile organic compound (VOC) signatures (Singletary et al.). The study evaluates the efficacy of a polymer-based training aid in training dogs to detect viral agents, successfully achieving discrimination between agent-based target odors and non-target biological agent-based odors. This highlights the potential of safely utilizing dogs as real-time, mobile detectors in surveillance and screening strategies.

The use of biomedical detection dogs during disease outbreaks, including the ongoing COVID-19 pandemic, is discussed in another article within this Research Topic (Maughan et al.). The authors outline the potential applications, capabilities, and limitations of biomedical detection dogs in disease outbreak scenarios. They also emphasize the need for inter-governmental cooperation and acceptance from the public health community to overcome barriers hindering the implementation of this valuable resource.

Lastly, a comprehensive review article summarizes current evidence and provides a general overview of the diverse aspects that may impact canine medical scent detection (Meller et al.). The experts provide recommendations for the future deployment of medical detection dogs, covering aspects such as the type of dogs, training paradigms, sample characteristics, biosecurity, safety considerations, and training and deployment scenarios. This article can serve as a reference point for the use of medical scent detection in disease control.

The inclusion of dogs as medical detection tools holds significant promise, particularly in the context of infectious diseases, highlighting the One Health approach (1), which recognizes the interconnections between human health, animal health, and the environment, promoting transdisciplinary collaboration. The articles presented in this Research Topic provide valuable insights into the capabilities of medical detection dogs, their potential in disease detection, and the challenges that must be addressed. With continued research and investment in olfactory sciences, future research needs to unlock the full potential of canine scent detection, paving the way for innovative and effective medical testing methods.

In summary, this Research Topic serves as a platform to critically examine the potential of canine medical scent detection, specifically in the context of infectious diseases like COVID-19. By consolidating current knowledge and fostering further research, there is a need to expand our understanding and harness the extraordinary olfactory abilities of canines for the advancement of medical diagnostics, ideally before the next pandemic strikes.

## Author contributions

HV: Conceptualization, Writing—original draft. GA: Writing—review and editing. A-LC: Writing—review and editing. DG: Writing—review and editing. FM: Writing—review and editing.
